# Autonomic dysfunction and its associations with clinical parameters in Familial Mediterranean Fever: a cross-sectional study

**DOI:** 10.1007/s00296-026-06076-6

**Published:** 2026-02-04

**Authors:** İmran Kalkan, Halise Hande Gezer, Mehmet Tuncay Duruöz

**Affiliations:** 1 Physical Medicine and Rehabilitation, Iskilip Atif Hoca State Hospital, Çorum, Türkiye, Turkey; 2 Physical Medicine and Rehabilitation Division, Rheumatology Subdivision, Marmara University School of Medicine, 34899 İstanbul, Türkiye, Turkey

**Keywords:** Familial Mediterranean Fever, Autonomic nervous system, Heart rate variability, Parasympathetic nervous system, Sympathetic nervous system, Inflammation, Quality of life

## Abstract

This study aimed to assess autonomic dysfunction in patients with Familial Mediterranean Fever (FMF), and to explore its association with disease characteristics and comorbidities. Thirty FMF patients and 30 healthy controls were enrolled in this cross-sectional study. Disease features and quality of life were recorded using the Familial Mediterranean Fever Quality of Life Scale (FMF-QoL). The Composite Autonomic Symptom Score-31 (COMPASS-31), Hospital Anxiety and Depression Scale (HADS), Fatigue Severity Scale (FSS), Jenkins Sleep Scale (JSS), and Fibromyalgia Rapid Screening Tool (FiRST) were noted. Parasympathetic nervous system was evaluated by R-R interval variability (RRIV) during normal and deep breathing and 30:15 ratio tests with electromyography, while the sympathetic nervous system was assessed by sympathetic skin response (SRR), blood pressure response to standing and sustained handgrip tests. FMF patients had higher total median COMPASS-31 scores than controls (15.09 vs. 5.12, *p* = 0.001), with significantly higher secretomotor, gastrointestinal, bladder, and pupillomotor subdomain scores (*p* < 0.05). RRIV during normal breathing was lower in the FMF group compared with controls (17.29 vs. 21.27, *p* = 0.015). No between-group differences were observed in sympathetic function tests (*p* > 0.05). In correlation analyses, RRIV during normal breathing correlated negatively with CRP (*r* = − 0.378, *p* = 0.04). Sympathetic parameters showed negative correlations with CRP (left plantar SSR latency: *r* = − 0.398, *p* = 0.03; blood pressure response to handgrip: *r* = − 0.405, *p* = 0.026) and with annual attack frequency (left plantar SSR amplitude: *r* = − 0.449, *p* = 0.01). Total COMPASS-31 scores correlated with FMF-QoL (*r* > 0.5, *p* < 0.001), HADS-anxiety (*r* = 0.550, *p* = 0.002), HADS-depression (*r* = 0.633, *p* < 0.001), FSS (*r* = 0.600, *p* < 0.001), JSS (*r* = 0.705, *p* < 0.001), and FiRST (*r* = 0.598, *p* < 0.001). FMF patients showed predominant parasympathetic dysfunction, while sympathetic findings were limited to correlations suggesting possible subclinical involvement. These autonomic alterations may contribute to reduced quality of life and associated symptoms such as fatigue, depression, and sleep disturbance.

**Supplementary Information:**

The online version contains supplementary material available at 10.1007/s00296-026-06076-6.

## Introduction

Familial Mediterranean Fever (FMF) is a prevalent inherited autoinflammatory condition, transmitted in an autosomal-recessive pattern and observed mainly in populations from the Eastern Mediterranean region [1]. The disorder is characterized by recurrent febrile episodes accompanied by serositis. Clinical manifestations may include chest pain, abdominal pain, arthritis, arthralgia and erysipelas-like erythema. Amyloidosis represents the most serious complication of FMF; however, its incidence has markedly declined compared with earlier decades. Colchicine remains the first-line therapy, and IL-1 inhibitors may be considered in cases of colchicine resistance or intolerance [2].

The autonomic nervous system (ANS) regulates multiple involuntary physiological functions and is composed of central and peripheral neural structures, including ganglia, plexuses, and nuclei.It regulates a variety of functions, including heart rate, blood pressure, gastrointestinal motility, secretory processes, urination, sweating, and thermoregulation. Anatomically, the ANS has a complex organization that spans multiple regions of the central and peripheral nervous systems, and its activity is modulated by central neurons responding to afferent stimuli [3]. The ANS comprises two major branches: the sympathetic nervous system (SNS) and the parasympathetic nervous system (PNS); the former facilitates energy expenditure, whereas the latter supports restorative and energy-conserving processes [4].

The autonomic nervous system and the immune system are closely interconnected through a bidirectional regulatory network. Autonomic imbalance can promote a pro-inflammatory state, while persistent inflammation and elevated cytokines can impair autonomic reflex pathways, particularly vagal modulation [5, 6]. Previous studies have demonstrated that autonomic nervous system dysfunction can occur in a variety of rheumatologic conditions [7–12]. However, results in FMF patients have been inconsistent, and autonomic function in this patient group has not previously been assessed using electrophysiological methods [13–20]. Importantly, objective electrophysiological assessments, such as RR interval variability (RRIV), the 30:15 ratio for parasympathetic activity, and sympathetic skin response (SRR) for sympathetic function have not been comprehensively applied to FMF populations, leaving a significant gap in the literature [21]. Given the chronic inflammatory burden of FMF and its known associations with fatigue, sleep disturbance, anxiety, depression, and fibromyalgia, autonomic nervous system dysfunction may represent an under-recognized mechanism contributing to these symptoms and to impaired quality of life [22, 23].

The aim of this study was to evaluate autonomic dysfunction in FMF by combining symptom-based tools with objective electrophysiological assessments and to compare these findings with those of healthy controls. The primary hypothesis was that parasympathetic function, assessed by RRIV during normal breathing (RRIV-NB), would be impaired in FMF patients compared with controls. All other autonomic measures, symptom scores, and correlation analyses were predefined as secondary and exploratory outcomes. A further secondary objective was to explore the relationships between autonomic parameters and clinical disease characteristics or comorbid conditions, including anxiety, depression, fatigue, sleep quality, and fibromyalgia.

## Methods

### Patient population

This cross-sectional study included 30 patients with a diagnosis of FMF who were being followed in the rheumatology outpatient clinic of a tertiary university hospital, along with 30 healthy controls, between March and October 2022. Ethical approval was obtained from the Marmara University institutional review board, and written informed consent was collected from all participants (Approval date and number: 09.2021.1427).

Patients aged 18–65 years who fulfilled the Tel Hashomer criteria for definite FMF were eligible for inclusion, as were healthy controls of the same age range without a history of systemic disease or regular medication use. Individuals with conditions or medications known to affect autonomic function, such as organ failure, diabetes mellitus, thyroid disorders, arrhythmias, pregnancy or lactation, peripheral neuropathy, or the use of agents including alpha-agonists/antagonists, beta-blockers, anticholinergics, calcium channel blockers, or angiotensin-converting enzyme inhibitors, were excluded from the study. A sample size calculation was performed using G*Power software. Based on previously reported heart rate variability values in FMF patients (29.8 ± 11.1) and healthy controls (44.2 ± 8.5), a minimum of 11 participants per group was required to detect a statistically significant difference with a power of 95% and a two-sided α level of 0.05 [17].

### Data collection

For all patients and healthy controls, age, sex, body mass index (BMI), educational status, and smoking status were recorded. In the FMF group, disease duration, family history, medications, colchicine dose, presence of amyloidosis, attack-related clinical features, number of attacks in the previous year, and MEFV mutation type identified by genetic analysis were documented. Disease activity was assessed using the Pras disease activity score, and quality of life was evaluated with the Familial Mediterranean Fever Quality of Life Scale (FMF-QoL). Laboratory parameters included erythrocyte sedimentation rate (ESR), and C-reactive protein (CRP). Inflammatory markers (CRP and ESR) were assessed on the same day as autonomic testing, and all patients were evaluated during an attack-free and clinically stable period.

The Familial Mediterranean Fever Quality of Life Scale (FMF-QoL) is a disease-specific tool consisting of 20 items scored from 0 to 4, producing a total score between 0 and 80; higher scores reflect poorer quality of life [23]. The Hospital Anxiety and Depression Scale (HADS) comprises 14 items divided into anxiety and depression subscales, each rated 0–3; scores ≥ 11 indicate clinically significant symptoms [24]. The Jenkins Sleep Scale (JSS) evaluates the frequency of four sleep-related symptoms. Items are rated on a 0–5 Likert scale, yielding a total score between 0 and 20, with higher scores indicating poorer sleep quality [25]. The Fatigue Severity Scale (FSS) consists of nine items rated from 1 to 7 that assess the impact of fatigue on daily functioning. The mean score is obtained by dividing the total score by nine; scores ≥ 4 indicate clinically relevant fatigue [26]. The Fibromyalgia Rapid Screening Tool (FiRST) comprises six yes/no items addressing core features of fibromyalgia. The total score ranges from 0 to 6, and scores of 5 or higher are considered suggestive of fibromyalgia [27]. The HADS, FSS, JSS, and FiRST questionnaires were applied only to the FMF group, as these instruments are designed to assess disease-related psychological and symptomatic burden.

### Assessment of the autonomic nervous system

Participants were asked to avoid strenuous exercise and caffeine and nicotine consumption before recording. Recordings were made during the day in a quiet, dimly lit, and well-ventilated room, with an empty bladder at least 2 h after the last meal. For the patient group, the interval between attacks was at least 1 week after the last attack.

The autonomic nervous system was evaluated using objective parasympathetic and sympathetic function tests, as well as the Composite Autonomic Symptom Score-31 (COMPASS-31). The COMPASS-31 questionnaire evaluates six autonomic domains incluiding orthostatic intolerance, vasomotor, secretomotor, gastrointestinal, bladder, and pupillomotor functions, with total scores ranging from 0 to 100 [28].

Parasympathetic function was evaluated through RRIV during NB, RRIV during deep breathing (RRIV-DB), and the 30:15 ratio using an EMG device (Medtronic Keypoint Portable, Denmark, 2007). Surface electrodes were positioned on the hands, and RRIV-NB was recorded in the supine position during spontaneous breathing over one minute. RRIV-DB was obtained during six deep breathing cycles performed over one minute. RRIV during both NB and DB was calculated using the formula: (maximum RR interval minus minimum RR interval) divided by the mean RR interval, multiplied by 100. The numerical difference and ratio between the two measurements were also documented. RRIV during NB and DB was analyzed as a continuous variable. Since universally accepted cut-off values for RRIV are not available and measurements are influenced by age and methodological factors, no dichotomous classification (normal/abnormal) was applied. To determine the 30:15 ratio, patients were recorded in a seated position for two minutes and then asked to stand up rapidly, with recordings continuing for an additional 50 s. The 30:15 ratio was calculated by dividing the longest RR interval occurring around the 30th beat by the shortest RR interval occurring around the 15th beat following standing. The reference values described by Ewing and colleagues were used for interpretation: values of 1.04 or higher were considered normal, values between 1.01 and 1.03 were considered borderline, and values below 1.01 were regarded as abnormal and indicative of parasympathetic dysfunction [29, 30].

Sympathetic function was assessed using SSR with an EMG device, the blood pressure response to standing, and the blood pressure response to sustained handgrip using a digitally calibrated sphygmomanometer and Jamar hand dynamometer. Sympathetic skin response recordings were obtained using surface electrodes, with measurements made from all four extremities. The low-frequency filter was set at 0.5 Hz, the high-frequency filter at 2 kHz, with a sensitivity of 0.5 mV/division and a sweep speed of 1 s/division. Electrical stimulation was delivered to the ipsilateral median nerve using 10–30 mA intensity and a pulse duration of 0.2 ms, and simultaneous recordings from upper and lower extremities were obtained. If no response was elicited, the stimulus intensity was increased in 10 mA increments up to a maximum of 50 mA. Participants who demonstrated no response to five stimuli at 50 mA were classified as non-responders. Latency was defined as the time from the stimulus artifact to the first deflection from the isoelectric baseline, and amplitude was measured peak-to-peak. The presence or absence of a response, as well as latency and amplitude values, were recorded [29]. To evaluate blood pressure response to standing, participants rested in the supine position for 10 min, then were instructed to stand up quickly and remain standing for three minutes. Blood pressure was measured immediately before standing and at the end of the 3-minute standing period using the right upper extremity. Systolic blood pressure reduction was assessed. A decrease of ≤ 10 mmHg was considered normal, 11–19 mmHg borderline, and ≥ 20 mmHg abnormal, indicating sympathetic dysfunction [29]. To assess blood pressure response to sustained handgrip, participants rested in a semi-recumbent position for five minutes. Baseline blood pressure was then measured from the non-dominant upper extremity. Using a Jamar dynamometer, maximal voluntary isometric contraction strength of the dominant hand was determined, and the handgrip test was performed at 30% of this value for five minutes. Blood pressure was recorded from the non-dominant arm at one-minute intervals throughout the maneuver. The increase in diastolic blood pressure was calculated as the difference between the highest diastolic value during the maneuver and the baseline measurement. According to the reference values described by Ewing et al., an increase of ≥ 16 mmHg was considered normal, 11–15 mmHg borderline, and ≤ 10 mmHg abnormal, indicating sympathetic dysfunction [29].

### Statistical analysis

Statistical analyses were performed with IBM SPSS Statistics 20. Normality of continuous variables was examined using the Kolmogorov–Smirnov test supported by histogram and Q–Q plot inspection. Categorical variables were summarized as frequencies and percentages; continuous variables as mean ± SD when normally distributed, and median (IQR) otherwise. Between-group comparisons used the chi-square test for categorical variables, the independent samples t-test for normally distributed continuous variables, and the Mann–Whitney U test for non-normally distributed variables. Correlations were assessed using Pearson or Spearman coefficients based on distribution. Correlation strength was classified as weak (< 0.40), moderate (0.40–0.60), or strong (> 0.60) [29]. Given the evaluation of multiple autonomic endpoints and several correlation analyses, no formal adjustment for multiple testing was applied. These analyses were considered exploratory and hypothesis-generating, and the results should be interpreted accordingly. A p-value < 0.05 was considered statistically significant.

## Results

The demographic characteristics of the FMF and healthy control groups were similar with respect to age, sex, BMI, and educational level (*p* > 0.05). The mean age was 34.53 ± 11.46 years in the FMF group and 34.10 ± 9.87 years in the control group. Eight patients with FMF (26.7%) and ten individuals in the control cohort (33.3%) reported current smoking (Table 1).

**Table 1:** Demographic characteristics of the FMF and control groups

	FMF Group (*N* = 30)	Control Group (*N* = 30)	*p*
Age, year	34.53 (11.46)	34.10 (9.87)	0.876
Sex, Female	13 (43.3%)	13 (43.3%)	1.000
Body mass index, kg/m²	24.46 (5.15)	25.40 (3.74)	0.425
Educational status			
Primary/middle school	3 (10%)	7 (23.3%)	0.356
High school	12 (40%)	9 (30%)	
University	15 (50%)	14 (46.7%)	
Smoking, yes	8 (26.7%)	10 (33.3%)	0.573

Data are presented as mean (SD) or n (%).

The clinical characteristics of the patients are summarized in Table 2. The mean disease duration was 175.03 ± 112.37 months. Twenty-eight patients were receiving colchicine alone, while two patients were treated with an IL-1 inhibitor in addition to colchicine due to high disease activity. The mean colchicine dose was 1.55 ± 0.46 mg/day, and the mean Pras disease activity score was 5.10 ± 1.56. Seventeen patients (56.7%) had low disease activity according to the Pras score, whereas thirteen (43.3%) had moderate disease activity. The mean number of attacks within the last year was 7.30 ± 9.22.

**Table 2:** Clinical characteristics of patients with FMF

Disease duration (months)	FMF Group (*N* = 30)
Treatments	175.03 (112.37)
Colchicine	28 (93.3%)
Colchicine + IL-1 inhibitors	2 (6.7%)
Colchicine dosage, mg	1.55 (0.46)
Pras disease activity score	5.10 (1.56)
Number of attacks in the last year	7.30 (9.22)
FMF Family History, yes	22 (73.3%)
Genetic mutation	
Yes	24 (80%)
No	0
Unknown	6 (20%)
Type of Genetic Mutation	
M694V Homozygous	3 (12.5%)
M694V Heterozygous	7 (29.2%)
M694V Compound Heterozygous	5 (20.8%)
Others	9 (37.7%)
ESH (mm/hr)	11.43 (8.81)
CRP (mg/L)	3.73 (4.91)
Disease characteristics	
Fever	25 (83.3%)
Abdominal pain	28 (93.3%)
Chest pain	12 (40%)
Arthritis	7 (23.3%)
Arthralgia	16 (53.3%)
Myalgia	11 (36.7%)
Erysipelas-like erythema	4 (13.3%)
FMF-QoL	28.96 (18.07)
JSS	7.6 (5.75)
HAD-A	13 (43.3%)
HAD-D	6 (20%)
FSS	20 (66.7%)
FiRST	4 (13.3%)

*ESR* Erythrocyte Sedimentation Rate,* CRP* C-Reactive Protein,* SII* Systemic Immune-Inflammation Index, * FMF-QoL* Familial Mediterranean Fever Quality of Life Scale,* HAD-A* The Hospital Anxiety and Depression Scale Anxiety,* HAD-D* The Hospital Anxiety and Depression Scale Depression,* JSS* The Jenkins Sleep Scale,* FSS* The Fatigue Severity Scale,* mg* milligram,* Mean* mean value,* SD* standard deviation, Data are presented as mean (SD) or n (%).

### Parasympathetic function tests between FMF and control groups

The RRIV-NB was significantly lower in the FMF group compared with the control group, whereas no significant differences were observed between the groups in the other parasympathetic function tests (Table 3).

**Table 3:** Parasympathetic function tests between FMF and control groups

	FMF Group (*N* = 30)	Control Group (*N* = 30)	*p*
RRIV-NB	17.29 (5.36)	21.27 (6.88)	**0.015**
RRIV-DB	34.64 (8.60)	36.80 (11.65)	0.416
DB-NB	17.35 (8.23)	15.53 (10.62)	0.462
DB/NB	2.12 (0.69)	1.79 (0.64)	0.060
30:15 ratio	1.20 (0.09)	1.23 (0.10)	0.333
30:15 ratio			
Normal	29 (96.7%)	29 (96.7%)	1.000
Borderline	1 (3.3%)	1 (3.3%)	
Pathologic	0	0	

*RRIV* R-R interval variability,* NB* Normal breathing,* DB* Deep breathing; Bold values indicate *p* < 0.05, Data are presented as n (%)

### Sympathetic function tests between FMF and control groups

No group differences were detected in SRR parameters or blood pressure–based tests. Latency and amplitude values from all four extremities were comparable between FMF patients and healthy controls (Table 4).

**Table 4:** Sympathetic function tests between FMF and control groups

SSR	FMF Group (*N* = 30)	Control Group (*N* = 30)	*p*
Right palmar latency, ms, mean (SD)	1588.53 (112.45)	1545.16 (115.86)	0.147
Left palmar latency, ms, mean (SD)	1561.13 (81.84)	1547.20 (110.98)	0.582
Right plantar latency, ms, median (IQR)	1969.50 (1915.75-2061.50)	2076.00 (1894.25-2174.50)	0.065
Left plantar latency, ms, median (IQR)	1996.83 (125.85)	2054.60 (152.52)	0.115
Right palmar amplitude, mV, median (IQR)	1.63 (0.89–2.60)	1.74 (1.02–3.55)	0.308
Left palmar amplitude, mV, median (IQR)	1.93 (111 − 3.20)	1.59 (1.14–3.52)	0.900
Right plantar amplitude, mV, mean (SD)	0.87 (0.47)	0.89 (0.59)	0.926
Left plantar amplitude, mV, mean (SD)	0.73 (0.54)	0.82 (0.42)	0.475
Blood pressure response to standing (SBP decrease), mmHg, median (IQR)	5 (8-3.75)	4 (6-2.75)	0.090
Blood pressure response to sustained handgrip (DBP increase), mmHg, mean (SD)	15.53 (3.52)	16 (3.11)	0.589

*SD* Standard deviation,* IQR* Interquartile range,* SSR* Sympathetic skin response,* DBP* Diastolic blood pressure,* SBP* Systolic blood pressure. Data are presented as mean (SD) or median (IQR)

### COMPASS-31 scores between FMF and control groups

The FMF group had higher total COMPASS-31 scores, as well as higher secretomotor, gastrointestinal, bladder, and pupillomotor subscores compared with controls (Table 5).

**Table 5:** COMPASS-31 test results in the FMF and control groups

	FMF Group (*N* = 30)	Control Group (*N* = 30)	*p*
Total COMPASS-31 score	15.09 (9.81–31.01)	5.12 (1.78–12.72)	0.001
Subdomains			
Orthostatic intolerance	0 (0–16)	0 (0–9)	0.138
Vasomotor	0 (0-1.66)	0 (0–0)	0.070
Secretomotor	4.28 (0-6.42)	0 (0–0)	**< 0.001**
GIS	6.78 (2.67–9.36)	1.78 (0-4.46)	**< 0.001**
Bladder	0 (0-2.22)	0 (0–0)	**0.003**
Pupillomotor	1.66 (0-2.41)	0.66 (0-1.33)	**0.029**

*COMPASS-31* Composite Autonomic Symptom Score-31,* GIS* Gastrointestinal system,* IQR* Interquartile range, Bold values indicate p < 0.05, Dara are presented as median (IQR).

### Correlations between autonomic tests and disease characteristics

Within the FMF cohort, the COMPASS-31 total score and all subdomains showed positive correlations with FMF-QoL, and the GIS subdomain correlated positively with Pras score, ESR, and CRP (Table 6).

**Table 6:** Correlation analysis between COMPASS-31 and disease characteristics

COMPASS-31		Age	Disease duration	Colchicine dosage	Number of attacks	Pras score	ESH	CRP	FMF-QoL
Total score	r	0.322	0.074	0.065	0.122	0.129	0.160	– 0.047	**0.568**
	p	0.083	0.697	0.731	0.520	0.497	0:397	0.805	**0.001**
Orthostatic	r	0.349	– 0.160	0.030	0.056	0.000	– 0.010	– 0.239	**0.437**
	p	0.059	0.398	0.876	0.767	0.999	0.958	0.204	**0.016**
Vasomotor	r	0.193	0.010	0.018	0.136	0.015	0.229	0.065	**0.451**
	p	0.306	0.959	0.925	0.475	0.939	0.224	0.732	**0.012**
Secretomotor	r	**0.397**	0.069	– 0.083	– 0.003	0.057	– 0.018	– 0.075	**0.600**
	p	**0.03**	0.717	0.664	0.988	0.765	0.925	0.695	**< 0.001**
GIS	r	0.023	0.240	0.344	0.327	**0.373**	**0.444**	**0.398**	**0.363**
	p	0.902	0.202	0.063	0.078	**0.042**	**0.014**	**0.030**	**0.048**
Bladder	r	**0.391**	– 0.115	– 0.037	0.280	0.059	0.320	0.083	**0.367**
	p	**0.03**	0.545	0.844	0.134	0.756	0.084	0.663	**0.046**
Pupillomotor	r	**0.437**	0.046	– 0.083	0.107	0.041	0.227	– 0.065	**0.432**
	p	**0.01**	0.811	0.664	0.572	0.830	0.227	0.733	**0.017**

*COMPASS-31* Composite Autonomic Symptom Score-31,* GIS* Gastrointestinal System,* ESR* Erythrocyte sedimentation rate,* CRP* C-reactive protein,* FMF-QoL* Familial Mediterranean Fever Quality of Life Scale, Bold values: Parameters with significant associations based on Spearman correlation analysis

COMPASS-31 total scores demonstrated a moderate correlation with HADS-anxiety (*r* = 0.550, *p* = 0.002) and a strong correlation with HADS-depression (*r* = 0.633, *p* < 0.001). COMPASS-31 scores were also strongly associated with fatigue (FSS; *r* = 0.600, *p* < 0.001) and sleep disturbance (JSS; *r* = 0.705, *p* < 0.001), and moderately associated with FiRST scores (*r* = 0.598, *p* < 0.001).

For parasympathetic measures, RRIV-NB correlated negatively with CRP (*r* = − 0.378, *p* = 0.04) (Table 7).

**Table 7:** Correlation analysis between parasympathetic function tests and disease characteristics

		Age	Disease duration	Colchicine dosage	Number of attacks	Pras score	ESH	CRP	FMF− QoL
RRIV-NB	r	− 0.281	− 0.223	− 0.033	− 0.103	0.020	− 0.352	**− 0.378**	− 0.041
	p	0.132	0.235	0.861	0.587	0.916	0.057	**0.040**	0.829
RRIV-DB	r	− 0.244	0.129	0.071	0.012	0.236	0.079	− 0.126	− 0.107
	p	0.195	0.495	0.708	0.948	0.209	0.677	0.508	0.573
DB-NB	r	− 0.071	0.281	0.020	0.021	0.234	0.180	− 0.053	− 0.085
	p	0.707	0.133	0.917	0.912	0.214	0.342	0.782	0.655
DB/NB	r	0.052	0.354	0.032	0.041	0.207	0.237	0.091	0.028
	p	0.786	0.055	0.865	0.831	0.273	0.206	0.633	0.884
30:15 ratio	r	− 0.124	0.140	− 0.033	− 0.022	0.224	− 0.018	− 0.318	− 0.270
	p	0.513	0.461	0.861	0.908	0.235	0.925	0.087	0.149

*RRIV* R-R interval variability,* NB* Normal breathing;* DB* Deep breathing,* ESR* Erythrocyte sedimentation rate,* CRP* C-reactive protein,* FMF-QoL* Familial Mediterranean Fever Quality of Life Scale, Bold values: Parameters with significant associations based on Spearman correlation analysis

For sympathetic tests, negative correlations were observed between CRP and left plantar SSR latency (*r* = − 0.398, *p* = 0.03), as well as between CRP and blood pressure response to sustained handgrip (*r* = − 0.405, *p* = 0.026). The amplitude of the left plantar SSR showed a negative correlation with the number of attacks in the preceding year (*r* = − 0.449, *p* = 0.01) (Table 8).

**Table 8:** Correlation analysis between sympathetic function tests and disease characteristics

		Age	Disease duration	Colchicine dosage	Number of attacks	Pras score	ESH	CRP	FMF-QoL
SSR right palmar latency	r	– 0.121	– 0.283	– 0.210	– 0.219	– 0.335	– 0.143	– 0.193	0.035
	p	0.526	0.130	0.264	0.245	0.070	0.451	0.308	0.854
SSR right palmar amplitude	r	0.000	0.131	0.134	– 0.066	– 0.015	– 0.069	– 0.149	0.242
	p	1.000	0.491	0.481	0.729	0.937	0.718	0.431	0.198
SSR left palmar latency	r	– 0.294	– 0.183	0.055	– 0.163	– 0.122	– 0.024	– 0.163	– 0.060
	p	0.115	0.333	0.774	0.389	0.521	0.901	0.389	0.751
SSR left palmar amplitude	r	– 0.053	0.182	0.000	– 0.083	0.052	– 0.094	– 0.141	– 0.156
	p	0.780	0.336	0.999	0.662	0.785	0.623	0.458	0.409
SSR right plantar latency	r	– 0.341	0.170	0.127	– 0.175	0.118	– 0.131	– 0.153	0.034
	p	0.065	0.370	0.505	0.354	0.534	0.489	0.419	0.859
SSR right plantar amplitude	r	0.101	– 0.028	0.065	– 0.213	– 0.062	– 0.069	– 0.163	0.197
	p	0.597	0.883	0.733	0.258	0.744	0.717	0.390	0.297
SSR left plantar latency	r	– 0.086	– 0.081	– 0.222	**– 0**.**449**	– 0.339	– 0.216	**– 0**.**398**	– 0.281
	p	0.651	0.671	0.237	**0**.**013**	0.067	0.251	**0**.**030**	0.132
SSR left plantar amplitude	r	– 0.149	– 0.038	– 0.001	– 0.183	0.211	– 0.098	– 0.346	– 0.313
	p	0.431	0.843	0.994	0.332	0.263	0.605	0.061	0.092
Blood pressure response to standing	r	0.064	0.040	– 0.073	– 0.073	0.032	0.036	– 0.207	– 0.347
	p	0.737	0.832	0.703	0.701	0.866	0.851	0.272	0.060
Blood pressure response to sustained handgrip	r	0.055	0.086	– 0.116	– 0.011	– 0.098	– 0.100	**– 0**.**405**	– 0.032
	p	0.773	0.653	0.540	0.952	0.607	0.597	**0**.**026**	0.866

*SSR* Sympathetic skin response.* ESR* Erythrocyte sedimentation rate,* CRP* C– reactive protein,* FMF-QoL* Familial Mediterranean Fever Quality of Life Scale, Bold values: Parameters with significant associations based on Spearman correlation analysis

## Discussion

In this study, we evaluated autonomic dysfunction both subjectively and objectively in FMF patients, and our findings suggest that patients with FMF may experience a higher burden of autonomic symptoms compared with healthy controls, particularly in secretomotor, GIS, bladder, and pupillomotor domains. The significantly reduced RRIV-NB suggests that parasympathetic impairment may accompany FMF even when other parasympathetic and sympathetic measures remain within normal limits. Furthermore, the positive associations between COMPASS-31 scores and clinical parameters, including FMF-related quality of life, inflammatory markers, fatigue, sleep quality, and depression measures, indicate a possible relationship between changes in autonomic measures is closely linked with disease activity and overall patient well-being.

FMF patients had substantially higher COMPASS-31 total and subdomain scores, including secretomotor, GIS, bladder function, and pupillomotor, than healthy controls. These results are consistent with the only prior study using COMPASS-31 in FMF by Moog et al., which found higher autonomic symptom burden and parasympathetic impairment on dynamic infrared pupillometry [19]. Furthermore, while autonomic bladder dysfunction has not previously been studied in FMF, the higher bladder function scores may suggest possible involvement, which should be confirmed with objective measures such as urodynamic investigations.

Decreased RRIV-NB in the FMF cohort was interpreted as a possible parasympathetic changes at rest. The absence of significant variation in RRIV-DB may indicate variability in individuals’ capacity to perform the maneuver consistently. The negative correlation between CRP and RRIV-NB, but not during deep breathing, supports the hypothesis that FMF-related inflammation specifically impacts this resting parasympathetic measure. In the literature, heart rate variability has been evaluated in FMF using various methodologies; however, no study has utilized electrophysiological techniques. Nussinovitch et al. found no significant variations in parasympathetic measures between patients and controls [14, 15], whereas another investigation indicated a substantial decrease in heart rate variabilility only in progressing amyloidosis [13]. Ardic et al. demonstrated delayed heart rate recovery and impaired vagal activation after exercise in FMF patients [18], whereas Canpolat et al. identified abnormalities in heart rate recovery, heart rate variability, and heart rate turbulence indices, even in the absence of cardiac symptoms [17]. Overall, these data indicate a possible susceptibility of parasympathetic function in FMF; nevertheless, variability in patient demographics, age, clinical and genotypic characteristics, and methodological differences likely account for the inconsistencies observed across studies.

To our knowledge, SSR parameters have not been previously investigated in FMF patients. In the present study, SSR latencies and amplitudes, as well as other sympathetic system assessments, including blood pressure changes during standing and sustained handgrip, did not differ significantly between the FMF group and healthy controls. Based on the data from our patient group, our findings did not demonstrate clear evidence of peripheral sympathetic sudomotor functions, which are primarily assessed by SSR. However, more specific assessment methods, such as the quantitative sudomotor axon reflex test or the thermoregulatory sweat test, may provide more detailed information. In addition, given the small patient population, the study may have insufficient power to detect moderate differences in sympathetic parameters. In the literature, Rozenbaum et al. reported orthostatic hypotension and postural tachycardia syndrome in 17–18% of FMF patients in two separate studies using the tilt-table test [31, 32]. In contrast, Moog et al. applied the cold pressor test, which has a similar mechanism and cutoff values to the sustained handgrip test, and found no significant differences compared with the control group [19]. The discrepancies between studies may be explained by the small sample size in our study, differences in the tests used to evaluate orthostatic intolerance, and heterogeneity in the clinical and genotypic characteristics of the patient populations.

Inflammatory markers were negatively correlated with autonomic test parameters in the FMF group. RRIV-NB decreased as CRP levels elevated, indicating that inflammation could affect cardiac vagal function, a correlation further supported by other studies associating increased CRP with reduced vagally mediated heart rate variability [33–35]. Likewise, decreased left plantar SSR latency was associated with increased attack frequency and CRP levels, suggesting a possible stimulatory influence of inflammation and disease activity on peripheral sympathetic sudomotor function; however, the lack of consistent results across all extremities restricts definitive interpretation. In addition to acute phase reactants, the correlation between attack frequency and plantar SSR latency may suggest a possible sympathetic involvement. Regulation of both parasympathetic and sympathetic changes in these patients may also be beneficial in controlling disease activity.

In the FMF patients, moderate correlations were observed between age and the COMPASS-31 secretomotor, bladder function, and pupillomotor domains, indicating that autonomic symptoms in these areas may increase with advancing age. Similar findings have been reported in previous studies, which likewise demonstrated that age-related autonomic symptom burden may increase in FMF patients [31]. We also found that the total COMPASS-31 score and all of its subdomains were positively associated with FMF-specific quality of life measures. Although no prior study has examined the relationship between autonomic symptoms and quality of life specifically in FMF, evidence from other systemic rheumatic diseases suggests that autonomic nervous system dysfunction is linked to impaired quality of life [36]. Furthermore, the GIS subdomain of COMPASS-31 showed significant positive correlations with Pras disease activity score, ESR, and CRP, supporting the notion that disease-related inflammation may contribute to autonomic symptom burden. Our findings also indicate that depression, anxiety, fatigue, poor sleep quality, and fibromyalgia are associated with autonomic symptoms in FMF.

This study has certain limitations, including the relatively small sample size and the fact that the patient cohort largely consisted of individuals with low-to-moderate disease activity, non-complicated disease, and low colchicine resistance. Given the number of autonomic endpoints and exploratory correlation analyses examined, some statistically significant findings may reflect chance variation; therefore, these results should be interpreted as exploratory and hypothesis-generating rather than confirmatory, particularly in the absence of multivariable adjustment due to the limited sample size. In addition, within the FMF cohort, higher levels of depression, fatigue, and sleep disturbance were associated with greater autonomic symptom burden; however, these findings reflect within-group associations and cannot be interpreted as FMF–control differences, as these PRO measures were not available in the control group. Nevertheless, the strengths of our study include the use of a healthy control group, the first electrophysiological evaluation of autonomic nervous system function in FMF, and the comprehensive assessment of the relationships between autonomic symptoms and signs with objective and subjective measures and clinical parameters.

## Conclusion

Our results suggest that patients with FMF may experience an increased autonomic symptom burden, which may indicate potential parasympathetic changes at rest, as evidenced by a decrease in RRIV-NB. In addition, these autonomic symptoms have been shown to have a negative effect on quality of life and to be associated with comorbid conditions, including fibromyalgia, depression, fatigue, sleep disturbance, and anxiety. Since cardiac autonomic dysfunction may contribute to morbidity and mortality, it is possible to evaluate it in FMF patients through simple, noninvasive electrophysiological assessments.

## Supplementary Information

Below is the link to the electronic supplementary material.

Supplementary Material 1.

## Data Availability

Data supporting the findings of this study are available from the corresponding author upon reasonable request. Data sharing complies with institutional and ethical guidelines.

## Declarations

### Related Congress abstract

Accepted at the ACR Congress 2023, Kalkan I, Duruöz MT, AB0032 ASSESSMENT OF AUTONOMIC DYSFUNCTION AND ITS RELATIONS WITH CLINICAL PARAMETERS IN FAMILIAL MEDITERRANEAN FEVER (FMF), Annals of the Rheumatic Diseases 2024;83:1241–1242. 10.1136/annrheumdis-2024-eular.4894.
